# Dissimilarity based Partial Least Squares (DPLS) for genomic prediction from SNPs

**DOI:** 10.1186/s12864-016-2651-0

**Published:** 2016-05-04

**Authors:** Priyanka Singh, Jasper Engel, Jeroen Jansen, Jorn de Haan, Lutgarde Maria Celina Buydens

**Affiliations:** Department of Bioinformatics, Genetwister Technologies B.V., Wageningen, The Netherlands; Radboud University Nijmegen, Institute for Molecules and Materials, Nijmegen, The Netherlands

**Keywords:** Bacterial wilt, Genomic prediction, Phenotype prediction, Genetic distance, Dissimilarity based Partial Least Squares

## Abstract

**Background:**

Genomic prediction (GP) allows breeders to select plants and animals based on their breeding potential for desirable traits, without lengthy and expensive field trials or progeny testing. We have proposed to use Dissimilarity-based Partial Least Squares (DPLS) for GP. As a case study, we use the DPLS approach to predict Bacterial wilt (BW) in tomatoes using SNPs as predictors. The DPLS approach was compared with the Genomic Best-Linear Unbiased Prediction (GBLUP) and single-SNP regression with SNP as a fixed effect to assess the performance of DPLS.

**Results:**

Eight genomic distance measures were used to quantify relationships between the tomato accessions from the SNPs. Subsequently, each of these distance measures was used to predict the BW using the DPLS prediction model. The DPLS model was found to be robust to the choice of distance measures; similar prediction performances were obtained for each distance measure. DPLS greatly outperformed the single-SNP regression approach, showing that BW is a comprehensive trait dependent on several loci. Next, the performance of the DPLS model was compared to that of GBLUP. Although GBLUP and DPLS are conceptually very different, the prediction quality (PQ) measured by DPLS models were similar to the prediction statistics obtained from GBLUP. A considerable advantage of DPLS is that the genotype-phenotype relationship can easily be visualized in a 2-D scatter plot. This so-called score-plot provides breeders an insight to select candidates for their future breeding program.

**Conclusions:**

DPLS is a highly appropriate method for GP. The model prediction performance was similar to the GBLUP and far better than the single-SNP approach. The proposed method can be used in combination with a wide range of genomic dissimilarity measures and genotype representations such as allele-count, haplotypes or allele-intensity values. Additionally, the data can be insightfully visualized by the DPLS model, allowing for selection of desirable candidates from the breeding experiments. In this study, we have assessed the DPLS performance on a single trait.

## Background

Genome wide association studies (GWAS) have been widely applied in human, plant and animal studies to identify genetic variants associated with complex traits [1–3]. In GWAS, the association between SNPs and a complex trait is usually analyzed by testing each marker individually. This requires a large number of significance tests. Because of this, a stringent *p-value* is generally used to select significant SNPs to reduce the number of false positive SNPs. At the same time, however, many real associated variants may be missed. The success of GWAS relies on the underlying trait architecture [4], heritability [5–7], effective population size and environmental factors [8, 9]. There is a general consensus that complex traits are controlled by many quantitative trait loci (QTL) with small effects. Typically, single marker GWAS analyses approaches are only able to capture QTLs with large effects and miss QTLs with small effects [1]. In addition, these significantly identified SNPs account for only a small fraction of the variation of the complex traits. Instead of GWAS where the target is to identify SNPs associated with a complex trait, one can also use SNPs to predict complex traits by fitting all SNPs simultaneously to select individuals. Prediction of a quantitative trait using all SNPs is referred as genomic prediction (GP) [10]. Breeders can use predicted trait values to select candidates for their future breeding programs, termed as genomic selection (GS) [11]. It has been shown that GP provides a cost-effective and time-efficient tool for breeders to predict traits, which may be difficult and expensive to measure directly, are limited to sex or only observable later in life. GP has been successfully applied in selection of breeding candidates in plants [12–14] and animals [15–17]. The approach has also been applied in humans to predict disease risk and many complex traits [1, 18]. Several studies have shown that the prediction accuracy of complex traits may be improved by using all SNPs simultaneously [1, 9, 12–15]. For example, Yang et al. [1] have shown that fitting all SNPs simultaneously leads to approximately ten-fold increase in the predictive ability of human height compared to the individual SNP.

The accurate prediction of a complex trait can be extremely challenging, as the trait may be affected by multiple loci that interact. Another major challenge is the fact that the number of SNPs in GP studies greatly exceeds the number of samples leading to the so-called ‘large-p - small-n’ problem. Because of this, many traditional statistical approaches are not applicable to such data. Deriving an accurate prediction of complex traits by the high-density SNPs, while at the same time taking into account possible interactions between multiple loci, requires powerful feature reduction methods. A variety of methods such as Bayesian regression [19–21], Genomic Best-Linear Unbiased Prediction (GBLUP) [19, 22, 23], kernel regression [24] and dimension reduction methods [25] have been developed and applied in GP.

Most of the suggested statistical models differ in their assumptions of the distribution of the SNPs effect. For example, the GBLUP model assumes that the SNPs effect size is drawn from a common Gaussian distribution and the variances of SNPs effect are equal. This unrealistic assumption of GBLUP corresponds to use of a single random effect term in the model, which is a severe and unnecessary limitation. Whereas the Bayesian methods [21, 26] assume that the variance of SNPs effect differs among loci with most of the SNPs having a zero to low effect and only a few having moderate to large effect. Several studies have shown that Bayesian regression outperformed GBLUP for the prediction of traits with few QTLs with large effect [4, 27, 28].

Multiple Linear Regression (MLR) is an often used quantitative technique for prediction from predictors [29, 30]. However, MLR can be applied for prediction when the number of independent variables does not significantly exceed the number of observations and no significant collinearity between predictors exists [31]. Considering the characteristics of genomic data, MLR is not directly applicable for GP. Partial Least Squares (PLS) [32, 33] may overcome these issues for high-dimensional and collinear data by combining the principles of Principal Component Analysis (PCA) and MLR. It has been successfully applied in metabolomics for analysis of high-dimensional chromatography, and mass spectrometry data [34, 35]. PLS tries to extract latent variables (LVs) that combine SNPs to optimally predict a dependent variable such as a complex trait, taking into account their mutual correlation. However, PLS cannot be directly used for SNPs, as these are generally discrete (often represented as 0, 1, 2 for bi-allelic SNPs) while conventional PLS has been developed for the analysis of continuous data.

Therefore, we propose to use Dissimilarity-based Partial Least Squares (DPLS) to predict one or multiple traits from a large set of SNPs. In DPLS, measurements of the dissimilarity between the accessions (instead of the raw SNPs) are used for prediction. Because of this, DPLS may also be used for GP, when the method is used in combination with a suitable measure of the genomic distance between genotype accessions. Note that, during the dissimilarity calculation (between accessions), SNPs information is lost, which means effect of SNPs on the traits cannot be directly calculated from DPLS. However, there is a pseudo-sample technique proposed in literature to extract variables interaction effect from DPLS model [36]. Unlikely PLS, which uses PCA-like technique to extract LVs, DPLS takes the advantages of Multi-Dimensional Scaling (MDS)-like technique to extract LVs to predict complex trait. Both PCA and MDS techniques are widely used for dimension reduction purposes. MDS uses a distance matrix and is often recommended to analyze distance matrices. MDS minimize the dimensions, while preserving actual distance between data points. The DPLS combines features of MDS and PLS in order to perform GP. Several measures have been developed and proposed in literature to calculate dissimilarity between genomic accessions from SNPs [37, 38]. As a case study, we have explored and compared eight of such widely used genomic distance measures (Table 1) in combination with DPLS to predict bacterial wilt (BW) in tomato. In this study we have used SNPs as predictors to predict BW. BW is a complex trait caused by bacteria *Ralstonia solanacearum* and is considered as one of the most destructive diseases for a wide range of crops, including tomato [39]. In this analysis we have not accounted for any environmental factor in the prediction model. We have focused this study on genotype effects, by comparing accessions grown in the same controlled greenhouse environment.

**Table 1:** The selected dissimilarity measures used to calculate genomic distance among tomato accessions from SNPs^a^

Distance	Equation	R-packages	References
Euclidean	${\mathbf{d}}_{\mathbf{i1i2}}=\sqrt{{\displaystyle \sum_{\mathbf{k}=\mathbf{1}}^{\mathbf{K}}{\left({\mathbf{x}}_{\mathbf{i1k}}-{\mathbf{x}}_{\mathbf{i2k}}\right)}^{\mathbf{2}}}}$	gstudio	[65]
Gower	${\mathbf{d}}_{\mathbf{i1i2}}=\frac{{\displaystyle {\sum}_{\mathbf{k}=\mathbf{1}}^{\mathbf{K}}}\;{\boldsymbol{\updelta}}_{\mathbf{i1i2}\mathbf{k}}\ast {\mathbf{d}}_{\mathbf{i1i2}\mathbf{k}}}{{\displaystyle {\sum}_{\mathbf{k}=\mathbf{1}}^{\mathbf{K}}}\;{\boldsymbol{\updelta}}_{\mathbf{i1i2}\mathbf{k}}}$ For nominal or factor variables **d**_**i1i2k**_ = **0**, (**if x**_**i1k**_ = **x**_**i2k**_) **d**_**i1i2k**_ = **1**, (**if x**_**i1k**_ ≠ **x**_**i2k**_)	daisy	[66]
Allele share	${\mathbf{D}}_{\boldsymbol{i}\mathbf{1}\boldsymbol{i}\mathbf{2}}=\frac{\mathbf{1}}{\mathbf{K}}{\displaystyle \sum_{\mathbf{k}=\mathbf{1}}^{\mathbf{K}}}{\mathbf{d}}_{\mathbf{i1i2}}\left(\mathbf{k}\right)$ Where **d**_**i1i2**_ **(k)** = {0, If individual **i**_**1**_ and **i**_**2**_ have two alleles in common at the **k** ^**th**^ locus, 1, If individual **i**_**1**_ and **i**_**2**_ have only single alleles in common at the **k** ^**th**^ locus, 2, If individual **i**_**1**_ and **i**_**2**_ have no alleles in common at the **k** ^**th**^ locus}	Custom-R-script	[67]
Nei	${\mathbf{d}}_{\mathbf{nei}}=-\mathbf{ln}\left[\frac{\left(\mathbf{2}\mathbf{N}-\mathbf{1}\right){\displaystyle {\sum}_{\mathbf{i}=\mathbf{1}}^{\mathbf{L}}}{\displaystyle {\sum}_{\mathbf{j}=\mathbf{1}}^{\mathbf{l}}}\;{\mathbf{p}}_{\mathbf{i}\mathbf{j},\mathbf{x}}{\mathbf{p}}_{\mathbf{i}\mathbf{j},\mathbf{y}}}{\sqrt{{\displaystyle {\sum}_{\mathbf{i}=\mathbf{1}}^{\mathbf{L}}}\left(\mathbf{2}\mathbf{N}{\displaystyle {\sum}_{\mathbf{j}=\mathbf{1}}^{\mathbf{l}}}\;{\mathbf{p}}_{\mathbf{i}\mathbf{j},\mathbf{x}}-1\right)}\left(\mathbf{2}\mathbf{N}{\displaystyle {\sum}_{\mathbf{j}=\mathbf{1}}^{\mathbf{l}}}\;{\mathbf{p}}_{\mathbf{i}\mathbf{j},\mathbf{y}}-\mathbf{1}\right)}\right]$ Where, the summation L is across loci and l is across alleles at each locus in population x and y (here individual)	gstudio	[68]
Bray	${\mathbf{d}}_{\mathbf{i1i2}} = \frac{{\displaystyle {\sum}_{\mathbf{k}=\mathbf{1}}^{\mathbf{K}}}\left|{\mathbf{x}}_{\mathbf{i1k}}-{\mathbf{x}}_{\mathbf{i2k}}\right|}{{\displaystyle {\sum}_{\mathbf{k}=\mathbf{1}}^{\mathbf{K}}}{\mathbf{x}}_{\mathbf{i1k}}+{\mathbf{x}}_{\mathbf{i2k}}}$	vegan	[69]
Jaccard	${\mathbf{d}}_{\mathbf{i1i2}}=\frac{\mathbf{2B}}{\left(\mathbf{1}+\mathbf{B}\right)}$	vegan	[70]
Kulczynski	${\mathbf{d}}_{\mathbf{i1i2}}=\mathbf{1}-\mathbf{0.5}\;*\;\left[\frac{{\displaystyle {\sum}_{\mathbf{k}=\mathbf{1}}^{\mathbf{K}}}\mathbf{min}\left({\mathbf{x}}_{\mathbf{i1k},}\ {\mathbf{x}}_{\mathbf{i2k}}\right)}{{\displaystyle {\sum}_{\mathbf{k}=\mathbf{1}}^{\mathbf{K}}}\;{\mathbf{x}}_{\mathbf{i1k}}}+\frac{{\displaystyle {\sum}_{\mathbf{k}=\mathbf{1}}^{\mathbf{K}}}\;\mathbf{min}\left({\mathbf{x}}_{\mathbf{i1k},}{\mathbf{x}}_{\mathbf{i2k}}\right)}{{\displaystyle {\sum}_{\mathbf{k}=\mathbf{1}}^{\mathbf{K}}}\;{\mathbf{x}}_{\mathbf{i2k}}}\right]$	vegan	[70]
GRM	$\mathbf{G}=\frac{\mathbf{ZZ}\boldsymbol{\hbox{'}}}{\mathbf{2}{\displaystyle \sum {\mathbf{p}}_{\mathbf{k}}\left(\mathbf{1}-{\mathbf{p}}_{\mathbf{k}}\right)}}$	Custom R-script	[22]

x_i1k_ and x_i2k_ = SNPs at locus k for accession x_i1_ and x_i2_ respectively

d_i1i2k_ = distance between i_1_ and i_2_ samples for SNPs at locus k

*B* Bray- Curtis dissimilarity

*G* Genomic relationship matrix

*Z* genotype information for all tomato accessions

*p*_*k*_ frequency of allele at locus k

^a^d_i1i2_ = distance between tomato accession i_1_ and i_2_

The prediction quality (PQ) of the DPLS model was measured in term of R2 estimated from observed and predicted trait values in a cross validation (CV) setup. Furthermore, we have compared the prediction performance of DPLS with a prediction based on SNPs found as significant in a Univariate association analysis (where SNP was used as fixed effect) and GBLUP (where SNP was used as random effect). We demonstrate GP with DPLS on a single trait. The method can however be applied to simultaneously predict multiple, possibly correlated traits.

## Results and discussion

The differences in information captured by various genomic distance measures for GP have not been explored. Therefore, we first explored the properties of the eight genomic distance measures of interest to this study by the Mantel test, heatmap visualization, and their application in Multi-Dimensional Scaling (MDS) before we studied their application in DPLS.

### Comparison of genomic information captured by different distance measures

#### Mantel correlation

The Mantel test was used to compare the relation between two distance matrices in terms of correlation (*r*) statistics. The pair-wise correlation results obtained from the comparison of genomic distance measures by the Mantel test are presented in Table 2. On the basis of the Mantel correlation statistics, the eight genomic distance measures can be grouped into two categories for the data investigated in this study. Any two genomic distance matrices, which show a Mantel’s test correlation > 0.70, are placed together in one group. The first group (hereafter Group-I) includes Euclidean, Gower, Nei and allele-share distance and the other four genomic distances i.e., Bray, Kulczynski, Jaccard and genomic relationship matrix (GRM) were placed in second group (hereafter Group-II).

**Table 2:**
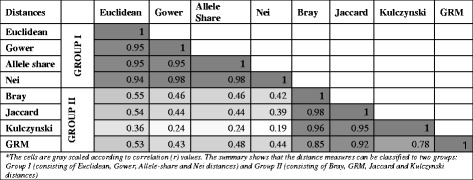
Summarized Mantel correlation statistics for analyzed genomic dissimilarity matrices*

#### Heatmap visualization

**Fig. 1:**
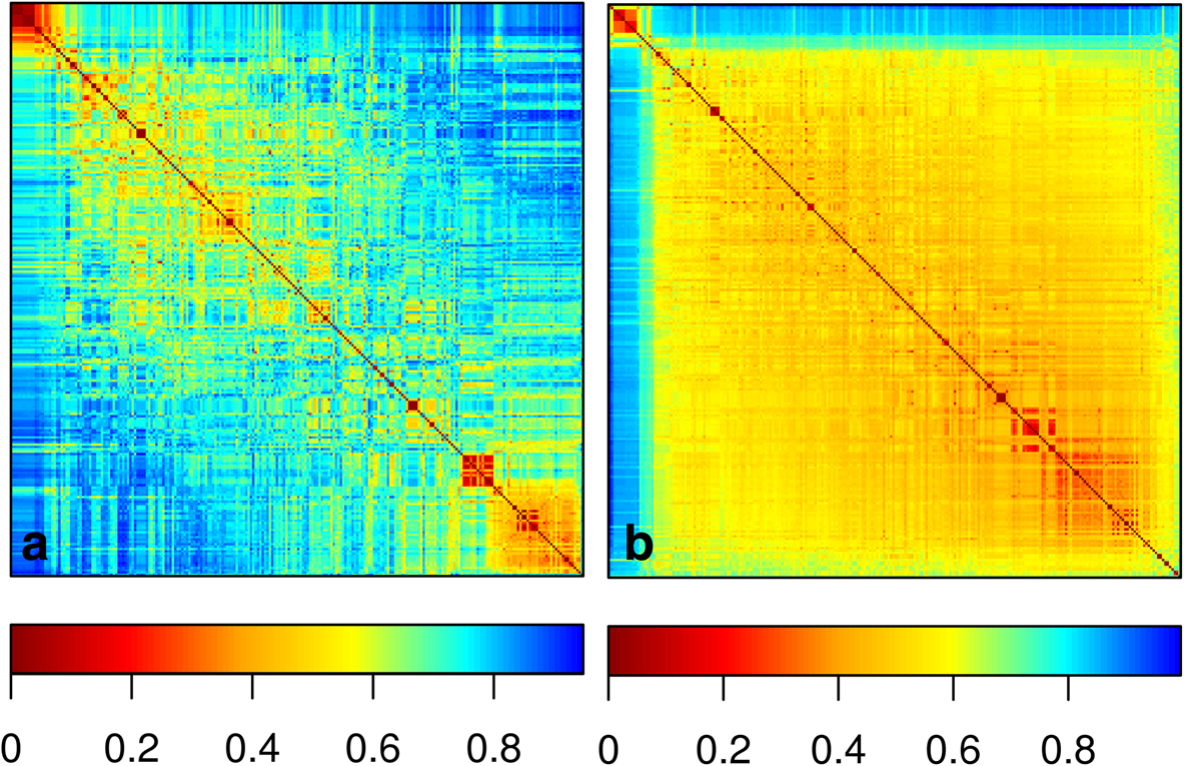
Heatmap representation of dissimilarity scores between 242 tomato accessions for Bray (**a**) and Euclidean (**b**). The pixels are colored in proportion to the genotypic dissimilarity between tomato accessions. Euclidean and Bray heatmap represents distance group-I and II respectively

In Fig. 1, the quantitative distance patterns between the 242 tomato accessions are visualized as a heatmap for the Euclidean and Bray distance. Both heatmaps show many sub-clusters of closely related tomato accessions. However, in contrast to the heatmap of the Euclidean distance (Group-I distance) the heatmap of the Bray distance (Group-II distance) shows many small clusters. The heatmap plot clearly shows that tomato accessions cluster differently in Euclidean and Bray distance space. In Fig. 1, the Euclidean and Bray distance were selected as representative distances of Groups-I and II identified by the Mantel test. Similar results were observed for the other distance measures within each group.

#### Multi-Dimensional Scaling (MDS) analysis

**Fig. 2:**
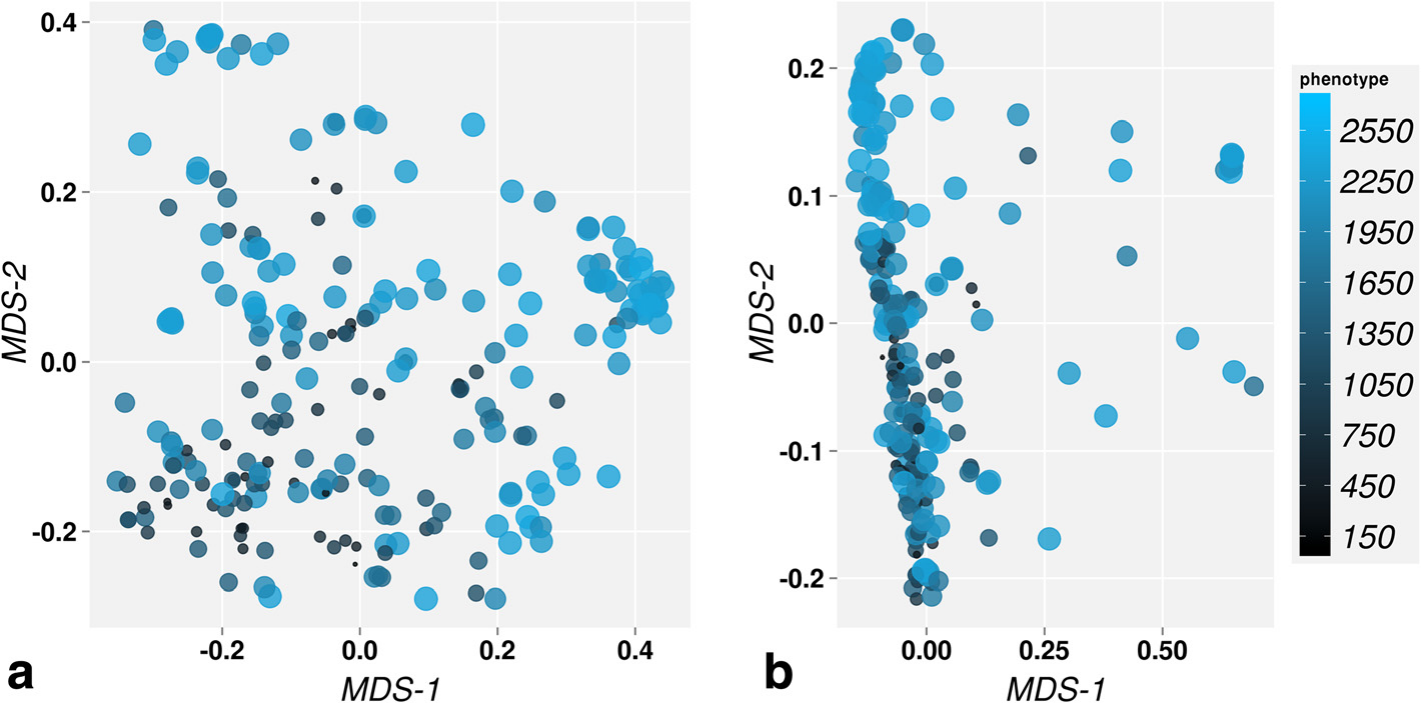
Multi-dimensional scaling (MDS) scores representation of Bray (**a**) and Euclidean (**b**) distances. MDS scores are visualized in first two dimension of MDS space, where MDS1 and MDS2 represents scores in first and second dimension respectively. The size and colors of bubbles are in proportion to the actual trait values (the measure of resistance against Bacterial wilt) of tomato accessions. The bigger bubble size represents higher resistance accessions

MDS models based on the two selected representative distance measures (i.e., Bray and Euclidean) were used to visualize the relations between the tomato accessions in a scatter plot (Fig. 2). The MDS plot of the Euclidean distance matrix suggests that most tomato accessions are genetically similar and form a big cluster with few smaller clusters of genetically less similar accessions. In the analogous representation of the Bray distances, tomato accessions were distributed throughout the entire plot with few accessions forming small clusters in MDS space.

In both plots the observed clusters do not clearly relate to the phenotype. We conclude that the two groups of distance metrics represent different structures within the genotype data, neither of which can be strongly associated to the phenotype measures by MDS. However, this insight could only be obtained from the MDS score plot that represents the relative differences and similarities between all accessions used in the study.

#### Phenotype prediction with DPLS

**Fig. 3:**
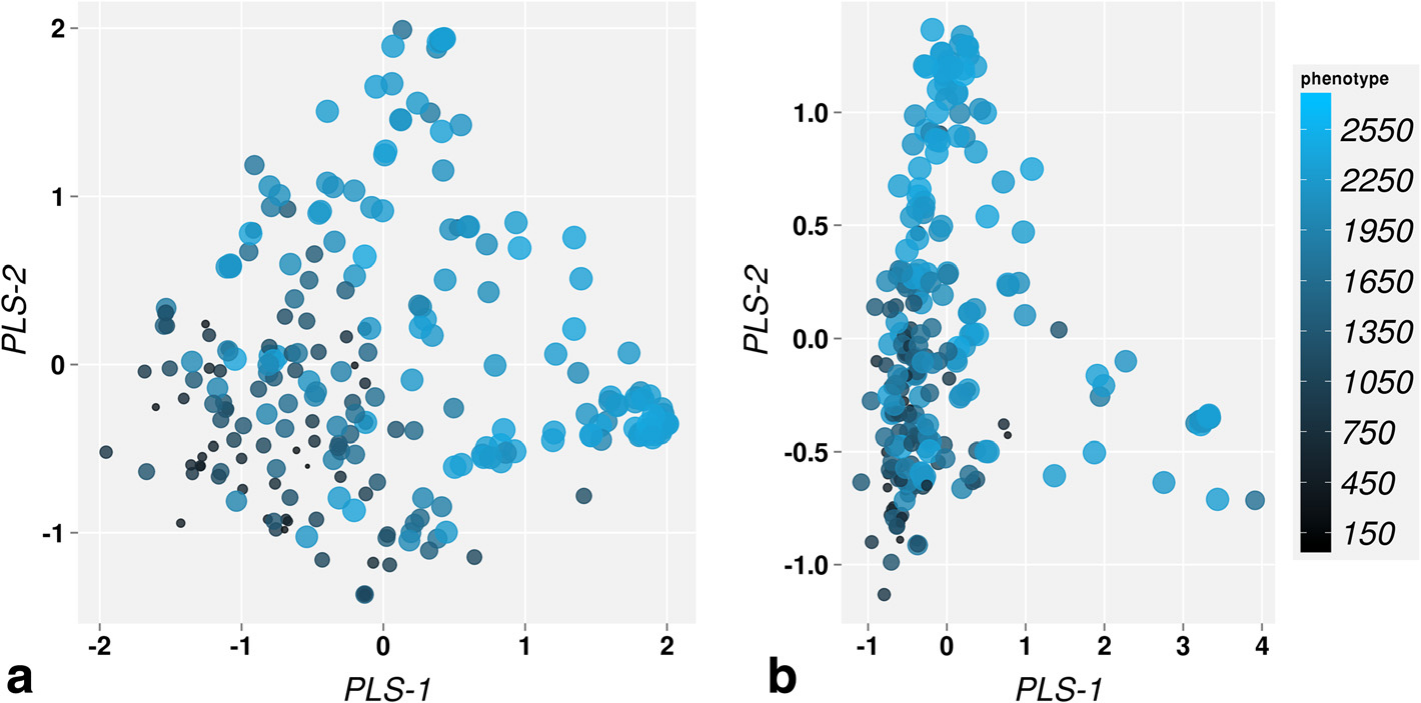
Dissimilarity based partial least squares (DPLS) scores representation for Bray (**a**) and Euclidean (**b**) distances. The DPLS scores are visualized in first two latent variables (LVs) where PLS1 and PLS2 present scores in first and second LVs space respectively. Each bubble represents a tomato accession. The size and color of the bubbles are corresponding to the actual trait values of tomato accessions where bigger size of the bubble corresponds to higher resistance accession. The PVE represents phenotypic variance explained by the DPLS prediction model

In this study, DPLS was used to relate genomic information captured by the distance measures indicated in Table 1 to the BW. For each distance measure repeated double cross validation (rDCV) (see Methods) was used to choose the optimal number of latent variables (LVs) to fit the DPLS model (see Table 3). As explained in the Methods section (equation 3) a big advantage of the DPLS method is that it also returns so-called score values for each accession. These scores represent the relative position of each accession in terms of their genomic distances associate to the trait values. As shown in Fig. 3, these scores can be visualized in a plot similar to the MDS analysis (Fig. 3), where large distances between accessions in the plot (the different dots) indicate large genomic differences. The score plots show a better arrangement of tomato accessions in the space of DPLS LVs when compared to the original data structure observed in exploratory analysis by MDS (see Fig. 2). The accessions are arranged according to their trait values; tomato accessions with similar trait values are close together in DPLS LVs space. The DPLS model is therefore better able to predict the trait values from the genotype dissimilarity scores data. Although the score plots of DPLS models based on different distance measures differ considerably, a direction within the space along which the trait value increases can be identified in both score plots.

**Table 3:** Dissimilarity based partial least squares (DPLS) prediction results over all dataset in a 10-fold CV setup

Distance	PQ^c^ (R^2d^)	RMSE^a^	Optimal LVs^b^
Euclidean	0.62 ± 0.005	370 ± 2.7	4
Gower	0.60 ± 0.0052	380 ± 2.8	6
Allele share	0.61 ± 0.005	380 ± 2.8	6
Nei	0.59 ± 0.005	390 ± 2.9	6
Bray	0.63 ± 0.004	370 ± 2.6	4
Jaccard	0.64 ± 0.0043	360 ± 2.8	4
Kulczynski	0.61 ± 0.0053	380 ± 2.8	4
GRM	0.62 ± 0.005	370 ± 2.9	5
GBLUP	0.61 ± 0.001	369.9 ± 0.66	NA

All the results presented in table are significant (with respect to *p*-value computed from permutation analysis). The results are averaged over 10-fold CV scheme. The 10-fold CV procedure was repeated 50 times. The standard error (se) calculated over 10-fold CV repetition. The last row present prediction results obtained from GBLUP. The PQ (R^2d^), RMSE and LVs represents prediction quality, root mean square error and latent variables respectively

^*a*^ *RMSE* stands for root mean square error

^b^ *LVs* stands for latent variables used for model building

^c^ *PQ* represent prediction quality

^d^ *R* ^*2*^ presented in the table are estimated for testset and not from training model. The value is calculated in a cross validation setup (some time indicated as Q^2^). This value is refer as prediction quality in this study

**Fig. 4:**
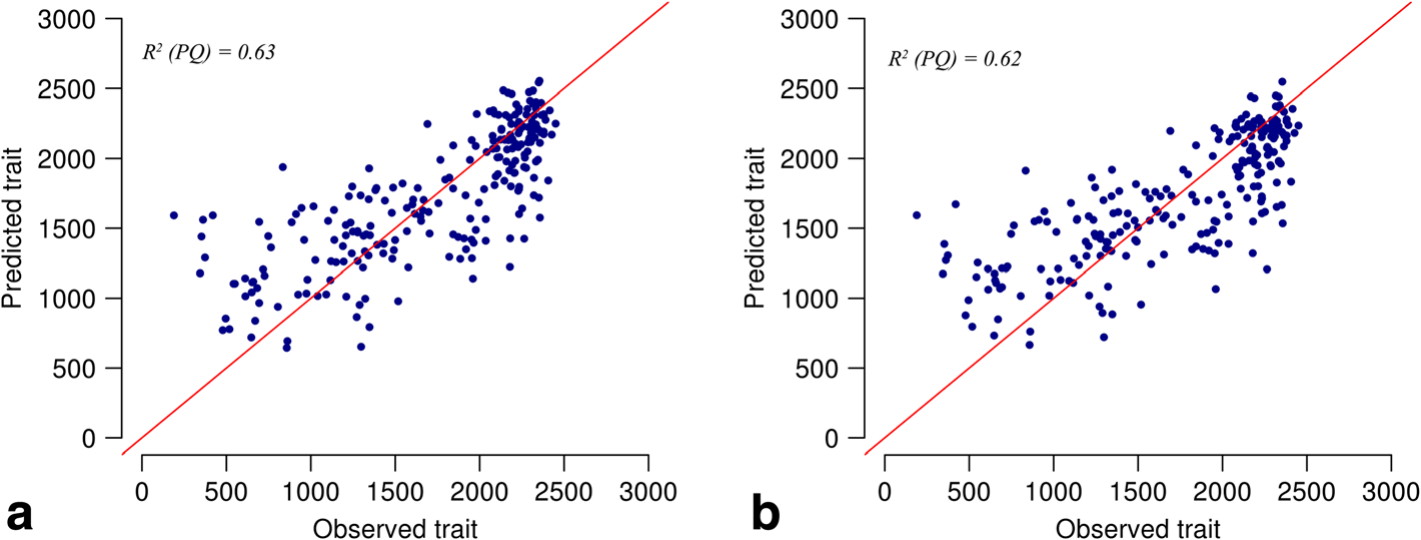
Dissimilarity based partial least squares (DPLS) prediction plot for Bray (**a**) and Euclidean (**b**). The prediction for each accession obtained in repeated 10-fold-CV scheme. Each point indicates mean value of accession prediction. Original and predicted value of BW traits are plotted on X and Y axis respectively. The R^2^ represent prediction quality and the red line indicates trend line for regression model

The DPLS prediction performance with respect to each distance measure is presented in Table 3. The performance statistics for each distance measure consist of PQ (i.e. R^2^ estimated on testset), model error measured in terms of Root Mean Square Error (RMSE) and the optimal number of LVs used for building prediction models. PQ range from 0.59 to 0.64 for analyzed genomic distance measures. The resulting RMSE was found to be similar for each genomic distance measure. These results (Table 3) indicate that the DPLS models may predict the trait similarly well between all distance measures for the studied BW-tomato data. The correlation (r) between measured and predicted BW values is visualized in Fig. 4 (hereafter prediction plot). The prediction plot shows a linear trend for the prediction based on both the distances (i.e., Bray and Euclidean). However, it seems from the prediction plot (Fig. 4) that accessions with higher BW values were predicted better than accessions with lower values. This follows from least-squares criterion used within DPLS, which it shares with conventional PLS, MLR and most other ‘conventional’ data analysis methods. This criterion gives more importance to the prediction of more distant accession. The heritability of the trait has also direct influence on GP. This is considered as theoretical upper limit for prediction accuracy and maximum variance explained due to genetic effects [8]. The estimated heritability for the BW is 0.76. The prediction results PQ (which is also a measure for variance explained in test set from prediction model) in Table 3, indicates that the variance explained by the DPLS model for the BW is close to the upper limit set by the estimated BW heritability.

#### Phenotype prediction with GBLUP

The performance of DPLS was compared to that of GBLUP in a 10-fold double cross-validation setup. The BW prediction results from the GBLUP model are indicated in Table 3. The trait can be predicted similarly well by GBLUP and DPLS. However, GBLUP does not provide any visual representation of the relations between individual accessions. A disadvantage of DPLS compared to GBLUP is that it does not provide information about SNPs effect directly. The genotype-based distance matrix from DPLS implies that information on individual SNP is lost during the modeling, which is retained by GBLUP. However, approaches such as pseudo-sampling [36, 40, 41] are available to interpret the effect of each individual SNP in GP. From this, the SNPs most relevant to the DPLS model can be obtained. However, Pseudo-sampling has not yet been applied to high-dimensional genomic data. Therefore this will be subject of a future study.

#### Phenotype prediction based on single SNP analysis

In previous studies using Univariate models for analysis of the BW-tomato data, 29 SNPs were found to be significantly associated with the studied BW (Unpublished). The phenotypic variance predicted from each SNP ranges from ~0.04 to ~50 %, which is lower than the variance explained by DPLS (59–64 %). This analysis clearly shows DPLS prediction has edge over single-SNP regression approach. The analysis clearly indicates that BW is a complex trait, which should be analyzed by multivariate methods that observe all SNPs within all accessions.

#### Advantages of DPLS as genomic prediction model

The results obtained from the proposed DPLS method indicate that its prediction performance is on par with that of GBLUP. Together with that DPLS provides some other beneficial characteristics. It can be applied to dataset of any dimension. DPLS reduces the dimensionality drastically and can handle missing values while computing distances or dissimilarities. It can handle the multi-colinearity that is omnipresent in genomic data and can be easily implemented using widely available software and methodology for conventional PLS. A major advantage of DPLS over other methods is the DPLS score plot, which represents arrangement of tomato accessions in DPLS space. This visualization provides a tool for the breeders to select the optimal candidates for their future breeding program. For instance, breeders can select the specific tomato accessions from the right panel of the score plot presented in Fig. 3, as candidates to specifically breed for a BW resistant tomato variety. This score plot based on the Bray distance shows that the first two LVs of DPLS explain about 56 % of variation in the phenotype. Additionally, the arrangement of tomato accessions with respect to the trait values in the plot shows that there seem to be at least two discrete groups of disease resistant accessions in the dataset. By inspecting this score plot, breeders can select candidates from both groups to breed for resistant varieties, to grasp more trait variability than with selection based on high resistant accessions. The score plot therefore enables, selection of multiple germplasm sources, which would be impossible if a single phenotype summary such as Estimated Breeding Values (EBVs) or other transformations of the trait. No existing method for GP provide such scores to compare individual accessions.

The other advantage of DPLS is that, it is flexible to various genotype representations. For Example, SNPs are usually encoded as discrete variables (i.e. 0, 1, 2 or 1, 0, −1) and many models uses this encoding as standard input for GP. The DPLS prediction model does not rely on such standard genotype representation since a distance matrix between accessions is used as input for the model. This makes it more flexible to data representation and may possibly be better applicable for GP in diploid or polyploid crops. The approach can also be very useful for analyzing complex phenotypes which are often collected in form of multiple traits to gather more information [42]. These traits are generally correlated and share a common genetic mechanism. The analysis of multiple traits together in a multivariate model may bring more power and increase chances of detecting SNPs, which have effect on individual or multiple traits [42, 43]. However, there is limited number of methods available, which can be applicable to multivariate trait analysis [44]. Successful prediction of multivariate responses with PLS has been reported in numerous references [45, 46]. We therefore expect that the DPLS may efficiently exploit the information from high-dimension SNPs to predict multiple potentially correlated traits. Assessing the DPLS performance on simultaneous prediction of multiple traits is a topic for future study.

## Conclusions

In this study DPLS, a novel approach for genomic prediction, is proposed for dealing with genomic data. This method employs the strengths of multivariate partial least squares (PLS) based prediction with the expression of genomic distances (calculated from SNPs) between individual accessions. This way, problems in the data such as the categorical nature of the variables, the large number of variables and their multi-collinearity are avoided. It was found that DPLS performs on par with GBLUP and better than Univariate prediction approach for GP. The prediction performance of the proposed method was close to the biologically imposed upper limit boundary set by the heritability of the trait. DPLS allows for visualization of the accessions with respect to the trait of interest, which may be invaluable for selection of specific candidates in agricultural breeding programmes.

## Methods

### Genotype

329 tomato breeding accessions were genotyped for 7321 SNPs using “SolCAP” array. The data was kindly provided by Bezo Zaden and East–west Seed. The SNPs are distributed across 12 chromosomes of tomato genome. A quality check (QC) was performed on the genotype data to exclude low quality SNPs and accessions from the analyses. SNPs were excluded if minor allele frequency (MAF) <0.01, proportion of missing value (PMV) > 0.10 or both. Tomato accessions with genotypes, but not phenotyped were excluded from the analyses. 242 tomato accessions and 6517 SNPs remains after the quality filtering and were used for GP.

### Phenotype

BW disease is caused by the *Ralstonia solanacearum* bacteria. It is one of the most destructive crop diseases in tropical, subtropical and some warm temperate regions of the world [39]. BW is a complex trait. In the current analysis, the trait was measured as percentage wilted plants at several time points (14, 21 and 28 days after disease inoculation, four replicates per accession), under greenhouse conditions. Based on the percentage-wilted plants, an area under disease progression curve (AUDPC) was calculated. Thus AUDPC is quantitative summarization of BW disease intensity over time. In this analysis we have focused on the interval between 0 and 28 days because measurements in this interval were available for most accessions. The AUDPC of the BW was calculated using equation below

$$ \mathbf{AUDPC}={\displaystyle \mathbf{\sum}_{\mathbf{i}=\mathbf{1}}^{\mathbf{n}}}\frac{\left({\mathbf{y}}_{\mathbf{i}}+{\mathbf{y}}_{\mathbf{i}+\mathbf{1}}\right)}{\mathbf{2}}\left({\mathbf{t}}_{\mathbf{i}+\mathbf{1}}-{\mathbf{t}}_{\mathbf{i}}\right) $$

Where **n =** total number of observation, **y**_**i**_ = percentage wilted plat at the **i**^**th**^ observation and **t** = time at the **i**^**th**^ observation.

### Genomic distance measurement

Eight dissimilarity measures (see Table 1) were explored and analyzed to find the most appropriate distance measure to use for genomic prediction. These measures were used to calculate distances between the tomato accessions based on SNPs. Furthermore, these calculated distances between tomato accessions were used in a DPLS model to predict BW. The evaluation of distances measures was based on the trait prediction accuracy for BW with the DPLS model. Additionally, initial exploratory analysis with the Mantel test [47, 48], heatmap visualization and MDS analysis were performed first on the distance measures to understand the correlation between the selected measures and their respective behaviors in the dataset.

### Exploratory analysis of distance measures

#### Mantel test

Relationships between distance matrices based on different genomic measures (see Table 1) were quantified using the Mantel test [48]. This statistical test constructs a linear comparison of two genomic distance matrices. The Mantel test first calculates the correlation between two distance matrices followed by a randomization procedure (permutation) to evaluate whether the observed correlation between two distance matrices is random or not [47, 49]. The Mantel test was performed with 1000 permutations, using the R package ade4 [50].

#### Heatmap visualization

Visual inspection of the distance matrices based on the measures in Table 1 was performed by plotting heatmaps of the genomic distances between the accessions within the BW-tomato data using the R-package ‘seriation’ [51].

#### Multi-Dimensional Scaling (MDS)

MDS is generally used to examine multivariate structure within a dataset by representing dissimilarity measures between specific accessions as distances in a much lower-dimensional space [52, 53]; we chose two dimensions for all MDS models. MDS analysis on genomic distance measures (see Table 1) was performed using the R-package ‘stats’ [54].

### Prediction model building and validation

#### Dissimilarity-based Partial Least Squares (DPLS)

The aim of this study is to determine if DPLS is a viable method for genomic prediction from whole genome marker data. The DPLS method employs the strengths of PLS based prediction with the expression of genomic distances (calculated from SNPs) between individual accessions. The general goal of PLS is to predict a set of response (dependent) variables**Y** from very large set of independent variables **X** (predictors), where for the BW- tomato data example **X** is the genotype matrix and **Y** is disease trait or response vector [55]. This prediction is achieved by first extracting a set of orthogonal factors called latent variables (LVs) from the predictors set. These latent variables (LVs) are considered to have best predictive power [31, 55]. The PLS model can be represented as

1 $$ \mathbf{X}=\mathbf{T}{\mathbf{P}}^{\mathbf{T}}+\mathbf{E} $$

2 $$ \mathbf{Y}=\mathbf{U}{\mathbf{C}}^{\mathbf{T}}+\mathbf{F} $$

Where,

**T** = **X**-factors scores (analogous to principal components in PCA although they are not the same)

3 $$ \mathbf{T}=\mathbf{X}\mathbf{W} $$

**U** = **Y**-factors scores

**P** = **X**-factors loadings and

**C** = **Y**-factors loadings

**E** and **F** = matrices of residuals

The regression coefficient that relates **X** to **Y** is obtained by:

4 $$ \mathbf{B}=\mathbf{W}{\left({\mathbf{P}}^{\mathbf{T}}\mathbf{W}\right)}^{-\mathbf{1}}{\mathbf{C}}^{\mathbf{T}} $$

Where,

**W** = **X**-factors weights i.e., projections of the objects of **X**-space onto **Y**-factor scores. The decomposition of **X** and **Y** are made so as to maximize the covariance between **T** and **U**.

In DPLS [56], the original data matrix **X** (genotype matrix) is replaced by dissimilarity matrix **D** (distance between tomato accessions). A summarized overview of genomic distance measures used to fit a DPLS prediction model in this study are given in Table 1. The DPLS model can be presented in following form.

5 $$ \mathbf{D}=\mathbf{T}{\mathbf{P}}^{\mathbf{T}}+\mathbf{E} $$

6 $$ \mathbf{Y}=\mathbf{U}{\mathbf{C}}^{\mathbf{T}}+\mathbf{F} $$

The **D** matrix is a square matrix. A double centering (i.e., subtracting row and column means of a distance matrix from its elements) has to be applied to matrix **D**. This means for DPLS the score matrix is same as the loading matrix (linear combination of predictors in **D** matrix), up to a scaling constanαt **α** to identify the model.

7 $$ \mathbf{T}=\boldsymbol{a}\mathbf{P} $$

The regression coefficient from the DPLS model can be calculated in similar equation as in classical PLS (see equation (4)). However, the regression coefficient **B** obtained from DPLS model is based on **D** matrix and not on **X** matrix as in PLS. The DPLS method also calculates scores for each accession. These scores are linear combinations of the predictor variables and are calculated in similar fashion as presented in equation (3). In DPLS, the predictors are the columns of the distance matrix, which contains the distance information on the samples in the dataset. The scores are calculated by using an appropriate weight matrix (**W**), which reflects the covariance structure between predictors and response variables. We have used the R-package ‘pls’ [57] and a custom script to perform DPLS for GP. Extensive validation methodology is available for PLS, that we adapt here for DPLS [58].

#### Repeated double cross validation (rDCV)

Three steps are critical when building a PLS/DPLS prediction model of 1) selection of optimal number of latent variables (LVs), and model building 2) the assessment of the overall model quality (or model reproducibility) 3) assessing significance of prediction model (model transportability).

**Fig. 5:**
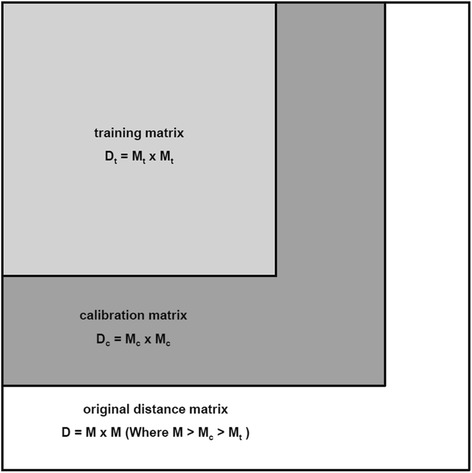
Illustration of distance matrix segmentation in double cross validation. Where D, D_c_ and D_t_ are squared distance matrix and represents distance scores between total accessions (M), accessions in calibration set (M_c_) and accessions in training set (M_t_) respectively

Several approaches including cross-validation (CV) setups are recommended for selection of LVs [58–60]. Here we used a so-called Double Cross-Validation (DCV) setup. In DCV [61], the data is first split into a test and calibration set. The calibration set is then further split into training and a validation set. In DPLS, the predictors are columns of the distance matrix, which contain the distance information on the samples in the dataset. The distance matrix is square, which requires specific splits for DCV. This matrix segmentation needs to be done in a specific fashion, to truly separate the information contained within them. The distance matrix is segmented such that the calibration training sets are square, as depicted in Fig. 5. The training and validation sets were used to determine the number of latent variables with optimal model error statistics. The error rate of the model with this optimal number of LVs to predict the phenotype was then determined by the test set. This procedure was repeated several times (in this case 50 times) hence in this study, we called this strategy as repeated-DCV (rDCV). The details concerning DCV/rDCV can be found elsewhere [58, 61].

#### Permutation analysis

Permutation analysis is often used for validation of PLS-based classification or regression [58, 61, 62]. Predictive multivariate models may be highly prone to overfit. Therefore it is common practice to assess their predictive performance against a benchmark where the effect of interest has been removed by randomization of the dependent variable, i.e. the trait value in this case. The model should not have any predictive power for such randomized data. A permutation test may be used to assess significance of reproducibility and transportability (prediction of new external validation set) of the model [63]. A permutation analysis was carried out in this study to validate the prediction accuracy observed with double-cross validation. The BW labels were permuted and randomly assigned to different accessions. A new DPLS model was then fitted to the permuted BW and the same model statistics were calculated. This procedure was repeated 5000 times and the model statistics were compared to the statistics obtained from the DPLS model on un-permuted labels. A *p*-value was obtained by combining all obtained statistics (mean and standard deviation RMSE) to assess the significance of difference between statistics (mean and standard deviation of RMSE) from original and permuted dataset.

### Conventional analysis methods

#### Genomic best linear unbiased prediction model (GBLUP)

Genomic BLUP [22] is considered a standard statistical method for genomic prediction. Several variations of BLUP models have been proposed in literatures for genomic prediction. We have used ridge regression best linear unbiased prediction (RR-BLUP) to predict BW t from SNPs. RR-BLUP assumes that all SNPs effect are normally distributed and have equal variance [28]. The model considered is:

$$ \mathbf{y}=\mathbf{1}\boldsymbol{\upmu } +\mathbf{Zg}+\mathbf{e} $$

Where, **y** is a vector of phenotype, μ is the overall mean, **Z** is design matrix corresponding to **g**, **g** is the vector of SNP effects and **e** is the vector of residuals. It was assumed that **g** ∼ **N**(**0**, **Gσ**_**g**_^**2**^) where **σ**_**g**_^**2**^ is additive genetic variance and **G**is genomic relationship matrix derived from SNP markers. These analyses were performed using the R-package rrBLUP [64].

#### Univariate analysis

In an unpublished study, 29 SNPs were found to be significantly associated with bacterial wilt s resistance. These SNPs were extracted from the BW-tomato data and fit in a univariate linear model. The SNPs were treated as fixed effect in this univariate model. The predictive ability (R^2^) was calculated for each individual SNP and compared with the R^2^ value obtained from multivariate approach DPLS.

## Ethics

No human or animal samples were used in this study. As far as applicable, all experiments have been performed according to legal guidelines.

## Consent to publish

The authors would like to thank Bejo Zaden and East-west Seed for access to the tomato dataset and to publish these results.

## Availability of data and materials

The distance matrices to perform genomic predictions can be provided upon request. The raw genotyping data cannot be shared due to the fact that the authors are not the owners of this data. Therefore the main contribution of this paper is on the methodological part of genomic prediction.

## Abbreviations

AUDPC: area under disease progression curve
DCV: double cross validation
DPLS: Dissimilarity based Partial Least Squares
GBLUP: genomic best linear unbiased prediction
GP: genomic prediction
GRM: genomic relationship matrix
GWAS: genome wide association studies
LVs: latent variables
MDS: Multi-Dimensional Scaling
PLS: Partial Least Squares
PQ: prediction quality
rDCV: repeated double cross validation
RMSE: root mean square error
SNP: single nucleotide polymorphism
